# Adaptation in adversity: innovative approaches to food security amidst COVID-19 in a remote First Nations community in Canada

**DOI:** 10.1186/s12889-024-21052-0

**Published:** 2024-12-18

**Authors:** Fatima Ahmed, Robert J. Moriarity, Nicholas D. Spence, Gisele Kataquapit, Celine Sutherland, Nadia A. Charania, Leonard J. S. Tsuji, Eric N. Liberda

**Affiliations:** 1 School of Occupational and Public Health, Faculty of Community Services, Toronto Metropolitan University, Toronto, ON M5B 2K3 Canada; 2 Department of Sociology, University of Toronto, Toronto, ON M1C 1A4 Canada; 3 Department of Health and Society, University of Toronto, Toronto, ON M1C 1A4 Canada; 4 Fort Albany First Nation, Fort Albany, ON P0L 1H0 Canada; 5 Department of Public Health, Auckland University of Technology, Auckland, New Zealand

**Keywords:** First Nations, Indigenous, COVID-19, Food security, Public health, Community-based participatory approach

## Abstract

The COVID-19 pandemic exacerbated food insecurity issues in geographically isolated communities, including Fort Albany First Nation (FAFN). This research examines FAFN’s adaptive strategies to improve food security, highlighting community resilience and leadership. Data were gathered through semi-structured interviews with 20 community members who were involved in the pandemic response, either as members of the pandemic committee or as managers of community programs. Thematic analysis revealed significant adaptation of existing programs and the establishment of new initiatives to address food security during the pandemic. Initiatives, such as the community garden and the Fort Albany Farmers Market were food security programs that existed prior to the pandemic, and despite labor shortages during the pandemic, logistical hurdles were addressed to maintain operations and enhance food distribution efficiency. New emergency food initiatives, backed by government support and community efforts, successfully delivered food to vulnerable households. Traditional subsistence activities, such as hunting and fishing, were essential for providing sustenance and strengthening community resilience. These adaptive strategies highlight the critical role of local leadership, community participation and ingenuity, and the utilization of Indigenous knowledge in overcoming food security challenges during crises. This paper underscores the need to support Indigenous food sovereignty and build resilient local food systems tailored to the unique needs of First Nations communities. The experiences of FAFN during the COVID-19 pandemic provide invaluable insights into the resilience and innovation required to improve food security in remote and vulnerable populations, emphasizing the need for sustained investment and policy support in these communities.

## Introduction

Many Indigenous communities in Canada have long faced challenges with food security, and the COVID-19 pandemic intensified these struggles, exacerbating inequalities and further restricting access to safe and nutritious food [1, 2]. In 2022, amidst the pandemic, 18% of families in Canada reported experiencing some level of food insecurity in the past year; however, this statistic is significantly underreported, as it does not include individuals living in the three territories, on reserves, or on other Indigenous settlements [3]. For Indigenous families, the situation was far more dire, with food insecurity rates significantly higher than the national average [4]. According to the 2022 Canadian Income Survey, Indigenous families above the poverty line experienced food insecurity at a rate of 31%, which is significantly higher than the 15% rate reported by non-Indigenous families [4]. Studies such as the First Nations Food, Nutrition and Environment Study, which assessed food and diet in 92 First Nations communities between 2008 and 2018, reported a high prevalence of food insecurity at 48% [5]. Several studies have cited factors such as, geographic location, the legacy of colonialism, lack of engagement in traditional activities and living circumstances (e.g., overcrowding), increasing the prevalence of food insecurity in First Nations households [1, 5–8]. During the pandemic, many of these issues were exacerbated and compounded with other stressors such as unemployment and poverty, further complicating access to safe and nutritious food for First Nations peoples [1].

The roots of food insecurity in Indigenous communities extend far beyond the immediate challenges of the pandemic, leaving many communities reliant on external food sources [9, 10]. Historical and current assimilative colonial laws (e.g., the Indian Act) and policies (e.g., the residential school system) have disrupted traditional food practices (e.g., hunting, fishing) and Indigenous governance structures [11, 12], exacerbating this dependence [13]. Additionally, treaties between “Indians” and the Government of Canada (on behalf of the British government) formally established “reserve” lands [14, 15]— often on lands unsuitable for agriculture—leading to food insecurity and reliance on government rations [16]. The banning of cultural practices, including feasts and sharing ceremonies, further disrupted the transmission of traditional knowledge related to food [13]. These restrictions undermined the ability for Indigenous communities to engage in subsistence practices, fostering dependence on government-controlled food systems.

Furthermore, the establishment of “reserves” in isolated geographic locations across Canada’s subarctic and arctic regions complicates access to remote Indigenous communities, many of which are only accessible by air or seasonal ice roads, significantly increasing the cost of food [7, 17]. The logistical difficulties and high transportation costs exacerbate food insecurity, making it challenging for these communities to obtain affordable and nutritious food options. According to the United Nations, food security is achieved when all individuals have both physical and economic access to safe, nutritious, and sufficient food to meet their dietary needs for an active and healthy life [18]. This concept is characterized by four key pillars: availability, accessibility, utilization, and stability [3, 6]. These pillars provide a framework for understanding the multifaceted nature of food insecurity. One example of this is the disparity in food security between rural and urban communities, where rural populations often consume higher quantities of processed foods due to challenges in accessing supplies [18]. The COVID-19 pandemic exacerbated these existing issues, due to disruptions in supply chains, increased costs, and limited economic access and availability to essential goods, amplifying the already precarious situation for food security in these communities [1]. Furthermore, socio-economic disparities such as higher rates of poverty and unemployment among First Nations communities in Canada also further restricts access to adequate food [1, 19]. Understanding the structural and underlying issues affecting food security, as well as the mechanisms that exacerbate these issues within vulnerable communities, is essential for developing effective interventions.

One community where these compounded issues have been particularly evident is Fort Albany First Nation (FAFN), a remote Cree community home to approximately 900 people, situated along the western shore of James Bay in subarctic Ontario, Canada [20]. As one of the Mushkegowuk Cree communities, FAFN is rich in traditions and deeply rooted in its connection to the land, water, and environment [21, 22]. However, colonialism and geographic isolation have long posed challenges to food security, leading to an increasing reliance on store-bought foods [8, 23–25]. Timmins, Ontario which is approximately 769 kms from FAFN is considered the main entry point for market food distribution year-round, and one of the closest cities with road access [26]. As a result, the community’s food supply is highly dependent on distant urban centers, further exacerbating issues of accessibility and affordability. One previous study indicated that the prevalence of household food insecurity in FAFN was approximately 70% [8]. The community is primarily accessible by air year-round, and by ice road during winter [27, 28]. FAFN faces ongoing challenges to securing food supplies, which come from sources such as farmers markets, grocery stores, community gardens, or local shops. While many community members continue to engage in traditional subsistence activities like hunting, trapping, and fishing [7], participation in these practices has declined in recent years [23, 24]. These challenges were further exacerbated by the COVID-19 pandemic, which disrupted supply chains and highlighted vulnerabilities in food access and availability.

As the pandemic unfolded in January 2020, several First Nations communities, including FAFN, began implementing travel restrictions and health screenings to protect the health and well-being of their members. These decisions are typically made by the Band Council, which is a governing body comprising of locally elected officials —the Chief and Council. In certain cases, as with FAFN, decisions are also made in collaboration with the Health Director. For example, Peetabeck Health Services, with the support of the FAFN Band Council, took proactive measures, including implementing symptom screenings for travelers arriving by air [20]. Alongside monitoring health measures, the community identified areas that would be impacted by the pandemic, from substance abuse issues to food security. However, without the infrastructure to purchase, transport, or store large amounts of food, FAFN and many remote First Nations communities were left in a more vulnerable position. Recognizing these vulnerabilities, the Government of Canada allocated $30 million through Indigenous Services Canada (ISC), a federal department responsible for policies related to Indigenous peoples in Canada, to support food security [1]. This funding aimed to provide immediate relief and improved access to essential food resources in Indigenous communities.

In response to the challenges identified and the additional government funding, FAFN adapted by strengthening its existing food sovereignty initiatives. Food sovereignty, which involves reclaiming decision-making power over the food system, was viewed by FAFN community members as a means to enhance independence, self-sufficiency, and develop new skills [26]. These efforts aimed to improve food security within the community, as highlighted in previous studies [26]. One key initiative since 2010 has been the community garden program, which focuses on cultivating local crops under ambient conditions, providing nutritious food for about 20 families. This program also empowers community members to establish their own home gardens by offering soil contaminant and nutrient testing, and consultations on crop selection and optimal planting locations, led by the community coordinator in collaboration with university researchers. Another pre-pandemic initiative is the Fort Albany Farmers Market, established in 2010. The market delivers fresh fruits, vegetables, meat, and other food products to the community every one to two weeks via chartered plane, depending on the season. It also supports programs like Meals on Wheels for Elders and a children’s snack and breakfast program, which resumed after pandemic restrictions eased.

During the pandemic, additional emergency initiatives were developed, supported by extra funding, including porch drop-offs for those isolated due to COVID-19 and food boxes created with support from local businesses. These efforts built upon pre-existing community programs and businesses to provide increased access to safe, nutritious and affordable food, and demonstrated the flexibility and responsiveness of FAFN’s food security initiatives in the face of new challenges. This paper explores the various strategies implemented by FAFN to adapt these initiatives to address food insecurity during the pandemic, examining how they were modified to meet the community’s evolving needs and the role of local leadership and community participation. By focusing on FAFN’s experiences, this paper highlights the resilience and innovation of Indigenous communities in times of crisis.

## Methods

This study, conducted in FAFN, stems from a longstanding partnership between the community and academic researchers. Community members (G.K., C.S.) and non-Indigenous researchers collaborated in previous studies over the last few decades to address various local health concerns [20, 29–32]. The present paper is part of a larger research initiative, put forward by the community, that evaluated the pandemic response in FAFN during COVID-19 with the aim to enhance their existing influenza pandemic plan in preparation for future pandemic responses. The study assessed community-implemented mitigation measures to reduce the impact of COVID-19 on healthcare systems and services, and to improve support services.

Central to this effort was the community’s pre-existing pandemic committee, which had been formed prior to the 2009 H1N1 influenza outbreak. This committee brought together various stakeholders, such as social services and police, to coordinate responses to pandemics [32]. The initial pandemic plan for the community was created during the H1N1 pandemic by community members (G.C.) and university researchers (N.C. & L.J.S.T.) [32–36]. Since the H1N1 pandemic, the health director has continued to maintain this group, holding annual meetings to review their pandemic plan. During the COVID-19 pandemic, any recommendations put forth which were not part of the pandemic plan were drafted and given to the Chief, who then consulted with their council before ratifying motions and approving the recommendations. Following the COVID-19 pandemic, community leaders recognized the need to update the plan based on their experiences and the new recommendations proposed during the pandemic.

This recognition informed the larger research effort, which was guided by a community-based approach where community members and researchers worked together in all phases of the research process—from conception, recruitment, and analysis to delivery—to ensure that the research was culturally relevant and met the community’s needs and priorities [37]. By employing this approach, we drew on multiple perspectives and a diverse range of community knowledge and experiences- to understand the development and implementation of innovative and contextually relevant solutions during the COVID-19 pandemic. From the larger study, food security emerged as a critical issue, showcasing the innovative solutions developed by community members in response to pandemic-related challenges.

### Study participants and data collection

Data for this research was gathered through semi-structured interviews with community members who played key roles during the COVID-19 pandemic. Participants included members of the pandemic committee and individuals, broadly referred to as program managers or coordinators, who were overseeing essential services/programs such as grocery stores, the food market, and the community garden. Potential interviewees were identified by the Health Director of Peetabeck Health Services and other leading members of the pandemic committee.

In total, 20 interviews (Pandemic Committee: 9; Program Managers: 11) were conducted in person from February to June 2023, adhering to COVID-19 safety guidelines set by the community. Each interview lasted between 30 minutes to 1 hour and were audio-recorded and transcribed verbatim. The interview questions were developed collaboratively with FAFN community leaders and the Health Director, covering various aspects of the COVID-19 experience. The questions focused on individual, community, and program-specific experiences during the pandemic, the implementation of public health measures, the vaccine roll-out, the long-term impacts of the pandemic, feedback on enhancing capacity for future pandemics, and the sources of information used to guide the response. Prior to participation, written informed consent was obtained from all interviewees, with measures in place to maintain confidentiality (i.e., removing personal identifiers and blurring photos). Data collection and handling of information followed OCAP^®^ (Ownership, Control, Access, and Possession) principles, ensuring data were collected, protected, used, and shared according to community guidelines [38]. This process was also directed by key community members who are authors of this paper. Ethics approval for the research was obtained from the research ethics boards of Toronto Metropolitan University (REB-2022-221), the University of Toronto (Protocol no. 00044366), and Auckland University of Technology (23/76).

### Data analysis

The 20 interviews were analyzed using a template approach to codebook thematic analysis, due to its structured framework [39]. This method involved familiarizing ourselves with the data through a meticulous review of the transcripts. Using NVivo^®^ computer software (Sage Publications Software, 2002), one author (F.A.) conducted an initial round of coding to generate preliminary codes based on recurring patterns and key insights from the data. These initial codes were then reviewed and refined collaboratively by three authors (F.A., L.J.S.T. & E.N.L.) who together developed a codebook that served as the foundation for further analysis.

The codebook was then applied to the data and was refined through ongoing discussions among the entire research team and community leaders to ensure that the codes accurately reflected the data. This process not only facilitated a deeper engagement with the data, but also helped to capture nuances in participants’ experiences, particularly around food security during the COVID-19 pandemic. By continuously revisiting and adjusting the coding template, we ensured a comprehensive representation of the data, validating and refining themes over multiple coding sessions [39].

One of the prominent themes which arose from this process was food security. To further analyze this theme, we adopted a hybrid approach, combining deductive and inductive coding techniques. Deductive coding was applied using pre-existing knowledge of community programs (e.g., grocery store, on-the-land programs, farmers market), while inductive coding allowed for the identification of new programs or initiatives that emerged. Knowledge of these programs and initiatives informed how the research team and community leaders (G.K. & C.S.) contextualized the emerging themes.

## Results

Out of the 20 interviews, food security was extensively discussed by 11 interviewees. Their perspectives are presented in the following sections, which highlight various pre-existing initiatives which were adapted, and new initiatives which were developed during the COVID-19 pandemic to address food security challenges within the community.

Existing challenges such as the geographical isolation of the community played an important role in exacerbating food security concerns during the pandemic. Typically, community members utilize the ice road, known as the James Bay winter road, during the winter months for travel where they can obtain non-perishable food items to use for the year. However, pandemic restrictions rendered this route inaccessible, with community members needing to seek special permission for travel. At the same time airflights into the community were also restricted, thus impacting the cost and availability of food, which became a pressing issue during the pandemic. Some participants spoke of these challenges:

*“I couldn’t wait for everybody to get vaccinated so we can get back to normalcy… When we had to go through those restrictions… We would run out of groceries. Families would need milk*,* you know*,* stuff like that. Those are the kind of things we went through”* (Participant 2).

*“I found that at the start it created panic on my clients because of the food situation… Thinking of ways to have food for people that caught COVID. It was hard*,* really hard at the start for people that caught COVID-19”* (Participant 16).

For these reasons, the community focused extensively on adapting existing programs and establishing new initiatives to address food security challenges, particularly among the most vulnerable members of the community.

### Being in the bush

During the COVID-19 pandemic, many community members in FAFN continued to engage in subsistence activities such as hunting, trapping, and fishing. Participants were encouraged to go to their bush (on-the-land) camps, traditionally located on family traplines. These gatherings allowed families from the community to continue sharing Indigenous knowledge, supporting both learning and cultural continuity. Additionally, even prior to the pandemic, the camps provided opportunities for the transfer of essential skills, such as hunting and trapping, which are directly linked to food security. Engaging in these activities during the pandemic provided essential food and significant mental health benefits by giving an opportunity for people to spend time on the land. One participant emphasized the positive impact of being outdoors, stating, *“It’s good for your mental health to get out for walks and stuff like that so the camp thing was great”* (Participant 3).

Another member of the pandemic committee highlighted the importance of being able to go in the bush during the pandemic stating, *“When you’re a treaty [rights holder] you can hunt and fish wherever you please*,* right… I said I’m not going to stop someone of their treaty right. Their inherent right to go hunt and fish… They [pandemic committee] started agreeing”* (Participant 7). For this reason, although initial lockdown measures required community members to stay home, and restricted any travel, the pandemic committee, along with Chief and Council, developed an initiative that allowed for community members to go out into the bush, with their family. This was also helpful in situations where overcrowding was an issue in homes, and allowed people to safely isolate, or maintain a distance at their bush camps. This was also an effective strategy during a spring flooding event, as one committee member explained,

*“That project that I did was called Oshemowin*,* meaning to get away… To leave the community to go to your camp… It was part of flood watch and also for COVID. Because they kind of overlap*,* right? Because at the time*,* it all happened like in the Spring*,* right? So that’s where we would probably be safer at the time because we were not in public”* (Participant 11).

This initiative was important for many families who relied on the land for traditional food, especially when store-bought goods became expensive and scarce during the pandemic. One program coordinator described the situation: *“Everything probably also got so much more expensive during the pandemic… We’ve been doing that*,* like getting our food from the land*,* you know*,* like the wild game out there… I always have those in my freezer”* (Participant 12). They also valued the communal aspect of sharing meals, stating, *“I like cooking… Then I like eating with people outside or even taking them to the camp. That’s where I take my food… We have goose sometimes or moose*,* rabbit. I have good memories over there”* (Participant 12). Being able to go out into the bush and maintain traditions provided a sense of normalcy and connection to culture.

The pandemic also impacted traditional practices and programs, particularly for the youth. As one program coordinator noted, *“That’s the impact on them [youth] from COVID… We used to do things for the youth. We took them out on the land*,* and when I was not working on the weekend*,* I would go out*,* take kids fishing or snaring and stuff and teach them how to do stuff in the bush*,* and that all just shut down*,* so we were not able to do anything”* (Participant 4). This disruption highlighted the importance of these activities in passing down traditional knowledge and maintaining cultural traditions.

Passing down knowledge and skills from Elders to younger generations was important for sustaining these traditions and adapting in times of crisis. One program coordinator shared her experiences: *“My mother was very resourceful… during that time [when I was living in Thunder Bay] she would have like income for one month and then we’d fall short like food-wise. And then she would take us out into the Bush*,* and we go fishing… Then we come back and have half our food there. That’s what she taught us… I always encourage my children to be prepared”* (Participant 12). This intergenerational transfer of knowledge ensured that traditional practices continued despite the challenges posed by the pandemic.

The emphasis on maintaining cultural practices and ensuring mental and physical well-being through connection to the land demonstrates the resilience and resourcefulness of the community during the challenging times of the COVID-19 pandemic.

### The adaptation of existing local food programs

#### Community garden

Initially established in 2010 as an adaptation to climate change, the garden has been integral to mitigating food insecurity and promoting food sovereignty. During the pandemic, the long-time community coordinator highlighted several challenges and adjustments that were necessary to sustain the garden’s operations.

**Fig. 1:**
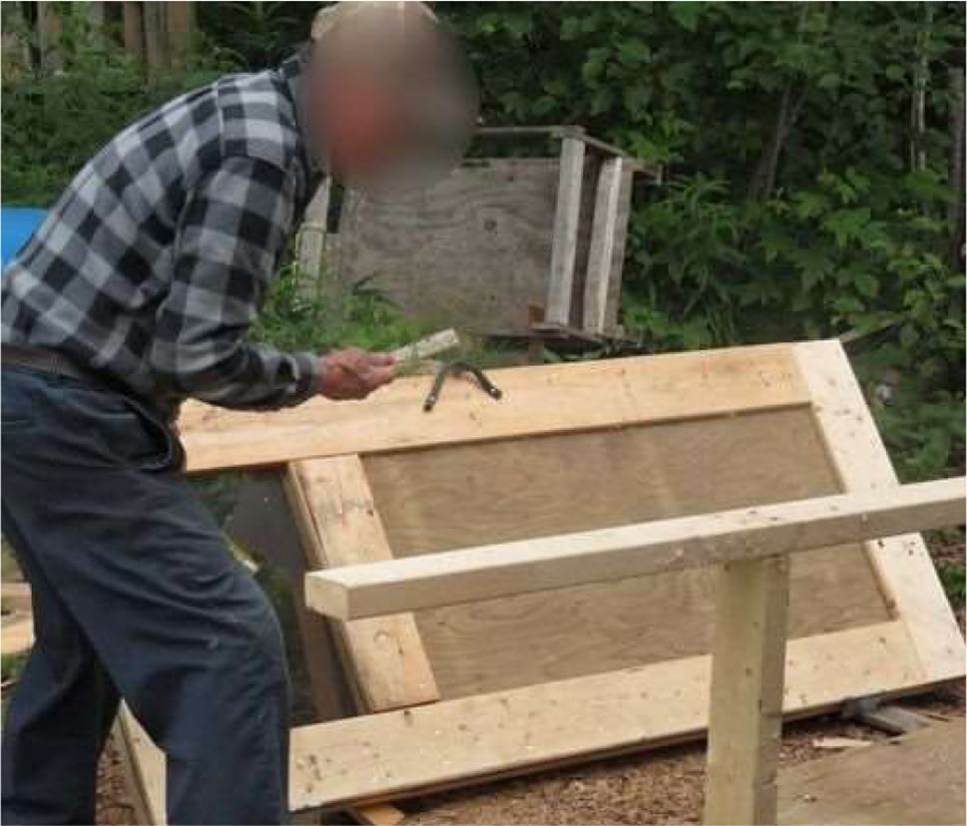
A photograph of an Elder working in the community garden to help build the lasagna garden during the growing season. (Photo Credit: Participant 10)

Prior to the pandemic, the community garden would employ between 4 and 5 community members to help maintain (and expand) the garden (Fig. 1.). However, during the pandemic, one of the biggest challenges was the lack of capacity needed to sustain the garden when some of the workers were isolating due to COVID-19. Similarly, additional infection control measures were difficult to implement within the project, *“It was kind of challenging…I had to tell most of my workers to keep your distance*,* wear your mask*,* but it’s hard to wear a mask when you’re working outside on a hot*,* sunny day”* (Participant 10).

They also emphasized the importance of self-isolation if symptoms appeared, which often left them working alone, *“Sometimes I was down to one worker*,* and I was down by myself. When everybody was sick*,* I just went to work*,* even though I didn’t feel too good myself*” (Participant 10). However, they found that being outside improved their health and mood during these times, adding,

*“I think being outside is better than being inside. There’s no air flow inside. But outside*,* you can smell the trees*,* the flowers. That’s where all your medicine comes from. I get my tamarack barks*,* cedar branches*,* and spearmint from the garden. There’s a lot of medicine out there; you just have to find it.”* (Participant 10)

Despite the challenges, the pandemic provided an opportunity for innovation in the garden. The coordinator experimented with different designs and crops, noting, “*I experimented on some stuff*,* and it turned out good. I did potatoes in the lasagna garden without soil*,* just compost and manure*,* and they grew more than the ones in the ground. This season*,* I want to try summer squash in the lasagna garden*” (Participant 10).

The community garden in FAFN has shown resilience and adaptability during the COVID-19 pandemic. It provided a critical source of fresh vegetables, and herbs while also serving as a space for health and well-being. The coordinator’s dedication and innovative approaches ensured the garden not only survived but thrived, underscoring the importance of local food production and community resilience in times of crisis.

#### Farmers market

During the COVID-19 pandemic, the market faced unprecedented challenges as the coordinator recalled, *“It was a lot more work than it had been*,* but food was one of the most important things that people needed during COVID”* (Participant 18). For this reason, the program required constant adaptation from the program coordinator and volunteers. One such adaptation was the shift to a contactless system for ordering and payment, which was a new process for many, “*We made lists and sent them out on the computer. People would check off what they wanted*,* and when we delivered the boxes*,* we had to put them down and run. The only way we could get payment was EMT (electronic money transfer)”* (Participant 18). This posed challenges for community members who faced financial constraints or difficulties with using technology, but community members worked together to work through these issues.

**Fig. 2:**
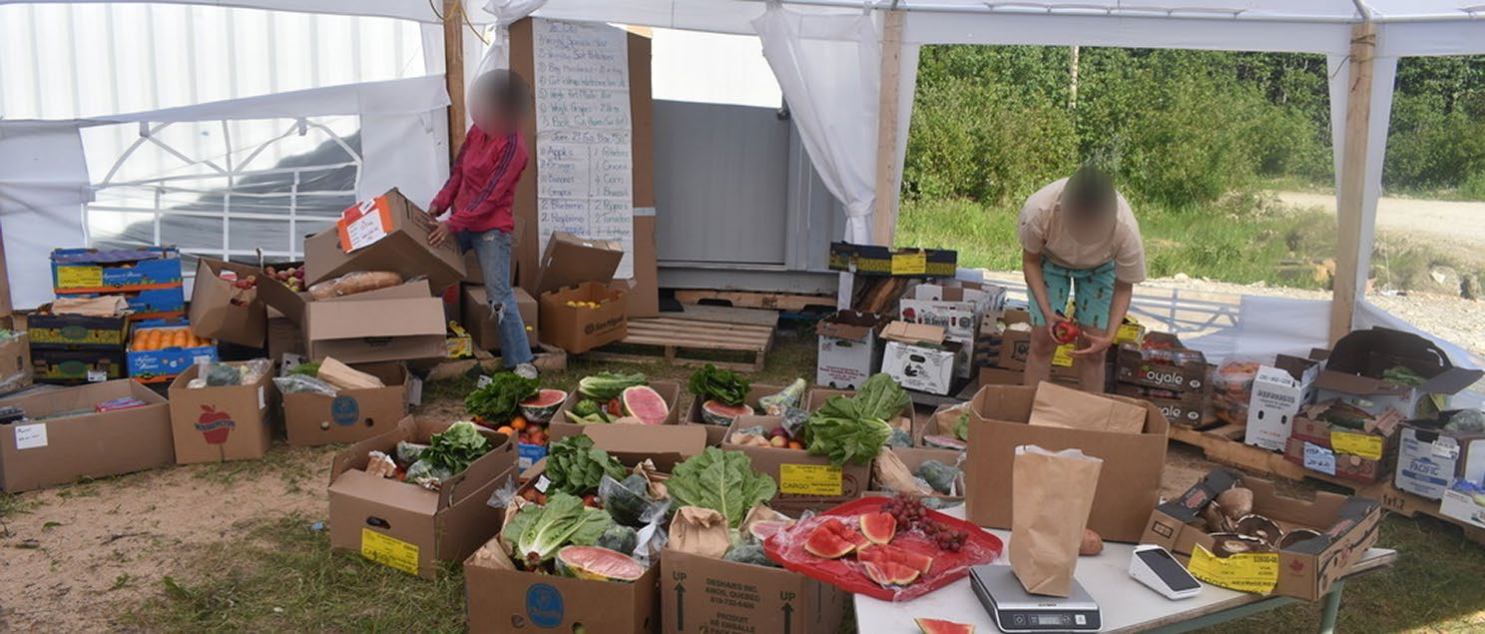
A photo of volunteers helping to set up food boxes during the COVID-19 pandemic in a tent outside during the summertime. (Photo Credit: Participant 18)

Additionally, the closure of the school gym due to lockdown restrictions, which acted as their primary storage and distribution location, provided more difficulties. As a result, curbside pickup was available from temporary locations such as a fire hall, the coordinator’s home, and a temporary outdoor wedding tent (Fig. 2.). However, each of these locations posed significant challenges.

One major issue, described by the coordinator, while using these temporary facilities was that there was no suitable location for leftover food storage,

*“So much of our food is perishable. We had to figure out where to store it before selling it again. We put a regular trapper’s tent up beside the storage unit and sold the food from there. We started curbside when we were allowed*,* thank goodness*,* because it was much less work for us. There was a lot of messaging*,* phone calls*,* and just organizing everything. Managing the food*,* making sure it was safe or that the veggies didn’t freeze or go bad because they were too hot in my house where we stored them”* (Participant 18).

Despite these setbacks, the market continued to adapt, providing approximately 20 Elders with boxes of food, which was delivered by home care workers.

**Fig. 3:**
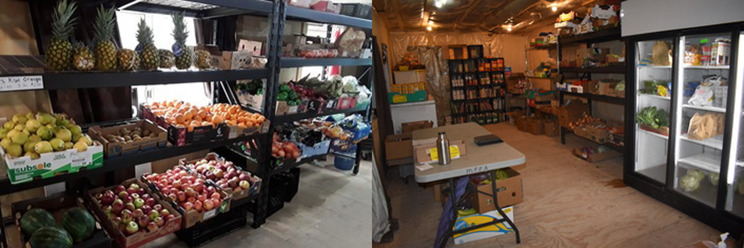
The permanent location of the farmers market set up with fridges and storage, which allowed for smoother operation during the COVID-19 pandemic. (Photo Credit: Participant 18)

The logistical challenges posed by the constantly changing locations of operation were immense, such as difficulties unloading planes and transporting food to various locations. Fortunately, the coordinator of the market was able to obtain additional funding during the pandemic to secure a permanent location (Fig. 3.).

This location became operational during the pandemic, with curbside pickup available while restrictions were ongoing, and in-person shopping available as restrictions eased.

**Fig. 4:**
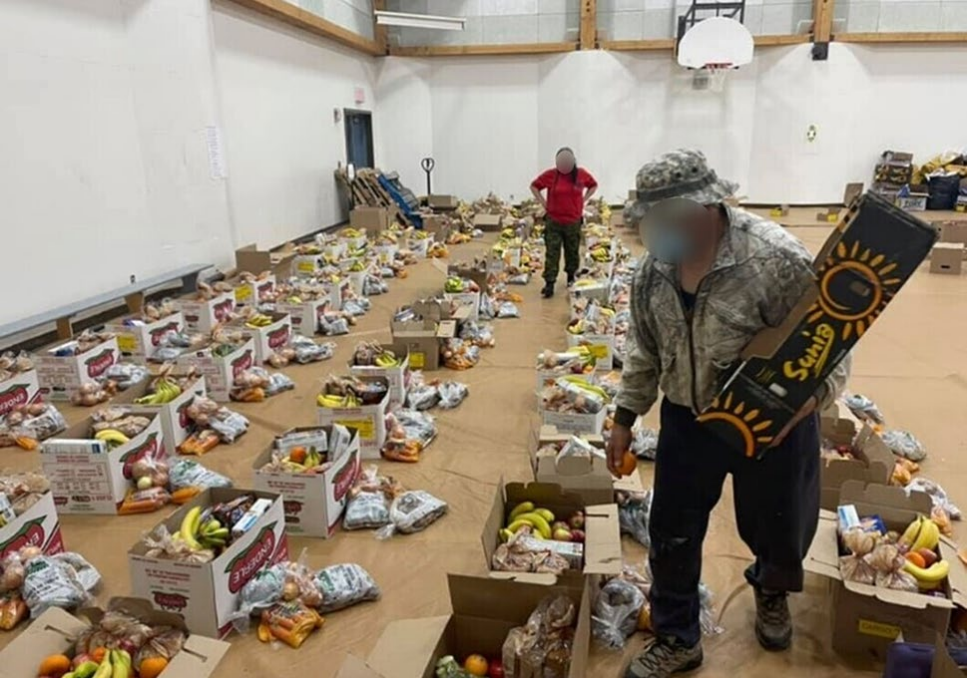
Two volunteers helping to assemble food boxes during the COVID-19 pandemic in the school. (Photo Credit: Participant 18)

The pandemic restrictions at times limited the number of volunteers allowed at the market, increasing the workload for coordinators and the few volunteers who could attend. At times, only two volunteers were available to run the market, significantly reducing its capacity (Fig. 4). Despite these challenges, volunteers remained dedicated. As the coordinator described, *“It was hard on a lot of volunteers that still tried to come and help us”* (Participant 18). Eventually, as restrictions eased, more volunteers were able to assist, increasing the market’s capacity, *“We ended up having to pack 80 to 90 boxes. I was so happy that we still had teachers and volunteer paramedics willing to help. We would load them onto our wagon and go through town to deliver them to everyone’s house”* (Participant 18).

Throughout the COVID-19 pandemic, the Farmers Market demonstrated the remarkable resilience and adaptability of the community-based program. The commitment to operating the market during unprecedented times in order to provide the community with safe, nutritious and accessible food was a significant achievement, as the coordinator described, *“We never stopped; not one week”* (Participant 18).

#### Emergency food initiatives

In addition to the challenges faced in maintaining the community garden and Farmer’s Market, grocers and convenience stores who supplied food for purchase in the community also had to adapt to the challenges brought on by the COVID-19 pandemic. For many of these stores, the constantly changing pandemic restrictions necessitated ongoing adjustments in operations. These efforts were coordinated through partnerships with various programs, businesses, and other initiatives. The Band Council, in collaboration with the pandemic committee, successfully secured financial support from the Government of Canada and Indigenous Services Canada (ISC) to fund emergency initiatives during the COVID-19 pandemic. As one participant explained, *“They [Canadian Government] gave us extra*,* the added cost because of*,* you know*,* we’re isolated and we’re further north*,* and things are more expensive. Food had to be flown in”* (Participant 8). This financial assistance enabled the community to sustain a high level of preparedness and respond effectively to the crisis. To access this funding, the Chief and Council maintained regular communication with the Government of Canada, providing updates on the local COVID-19 situation and its impact on the community. Representatives from ISC played an active role in supporting the community’s efforts, as one participant described, “*They [ISC] put the proposal together for me and said*,* ‘Here*,* we’ll give you this funding’. Then I told my team*,* ‘We’ve got some money; we can cover all those things [food costs].’ It helped*,* eh?”* (Participant 9). With the additional funding from the Government of Canada, the community was able to invest in, and continue supporting, essential programs and support services to address the challenges brought on by the pandemic.

**Fig. 5:**
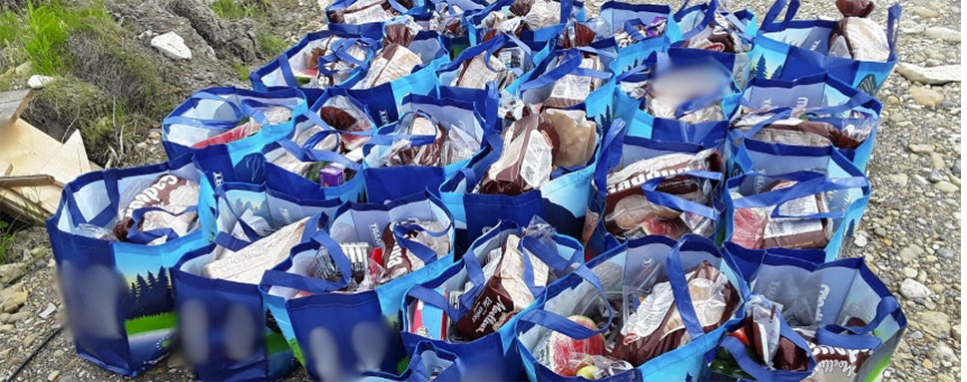
The delivery bags which were prepared through a local store in the community and delivered to those who were isolating at home because of COVID-19. (Photo Credit: Participant 18)

Despite these efforts, for some local businesses that supply food and other essential products, the constantly changing pandemic restrictions significantly affected their sales. One employee described the impact, *“We had to shut down for maybe two weeks. We were just doing curbside pickup*,* and that really affected the sales…we also did home deliveries”* (Participant 2). Although curbside pickup and home delivery methods were offered, the organization had to temporarily close its doors to manage the situation more effectively. An employee elaborated on these changes, saying,

*“We closed the doors for a while there because it was too hectic… When the (Canadian) Rangers came*,* they helped. We did the phone calls*,* the emails… get the groceries for the customers. Put the list down*,* shop for them*,* and buy them with just their name on them*,* and the Rangers just delivered (*Fig. 5*) … It was hectic… We all decided that we should do that rather than risk ourselves getting COVID because it (COVID cases) was really high at that time. I think at one point we had 70 some cases…and I don’t know how many close contacts at that time”* (Participant 6).

These measures also impacted various community-based programs’ ability to provide and obtain traditional foods. A program coordinator explained, *“Some programs that help provide traditional food to people in the community were shut down…we already had purchase orders in place for our program*,* and then suddenly everything stopped”* (Participant 4). This abrupt halt in programming required quick adjustments and rethinking how to meet the community’s food needs.

One approach was to identify the most vulnerable in the community, including those who contracted COVID-19, and understand their food security needs during this time. Coordination of these initiatives was successful, largely due to the involvement of members of the pandemic committee, volunteers, Canadian Rangers, and other community leaders. The Canadian Rangers, a component of the Canadian Armed Forces Reserves, operate in remote regions of Canada, with many members being Indigenous peoples from their respective communities. They provide vital support to these communities and are on standby in the event of emergencies, such as, search and rescue operations, or other forms of assistance. The sergeant of the Rangers, who resides in the community, collaborated with the Chief and Council, as well as the pandemic committee, to ensure the immediate deployment of Rangers when needed. As one participant described,

*“As a Ranger*,* you got to go out there and make sure everything’s well. Everybody’s looked after. So*,* then we had to do a lot of coordination in that process… I’m going to need support from Fort Albany First Nation*,* to say OK I need you to release the Rangers…Go to the airport*,* pick up the supplies*,* drop them off to school*,* separate the supplies*,* go door to door*,* drop off all the supplies*,* and keep and keep doing it again”* (Participant 10)

**Fig. 6:**
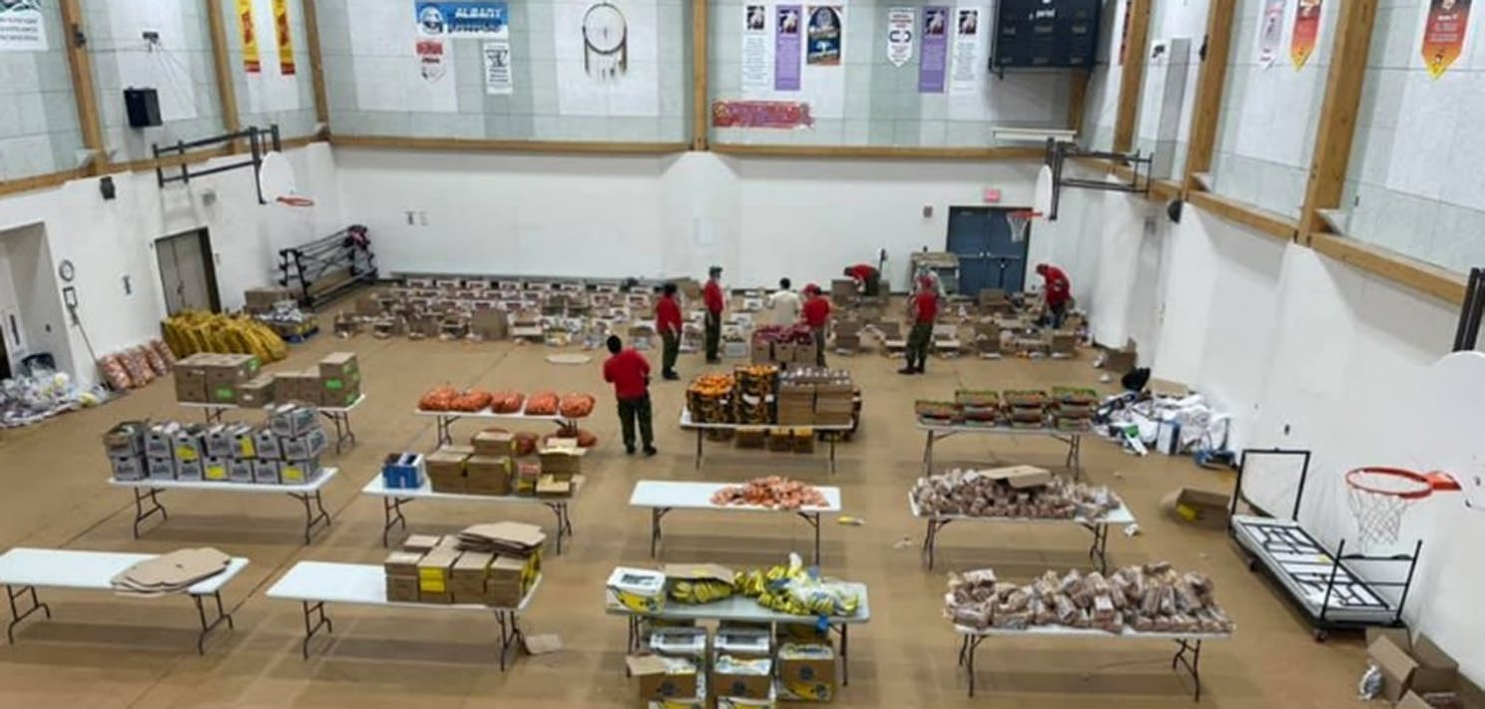
The Canadian Rangers putting together food packages in the community gym. (Photo Credit: Anonymous Canadian Ranger)

Their involvement was important to various community organizations and programs, particularly in coordinating and delivering safe and nutritious food. As one participant explained, *“We organized food hampers with the Rangers (*Fig. 6*). They handled delivery and everything”* (Participant 11).

Although their role in delivering groceries was important, it came with its own risks, *“At one point*,* the Rangers even contracted COVID during deliveries… It was a challenging time”* (Participant 6). These risks at times required multifaceted roles to be taken on by community members, *“we had to play the double role… Going to the airport*,* picking up supplies*,* dropping them off at school*,* separating the supplies*,* going door to door*,* and repeating the process”* (Participant 8). The involvement of various community members and organizations underscored the collective effort required to sustain these initiatives.

The coordination and dedication of these efforts were crucial in maintaining community safety and ensuring that those in need received support. Individual volunteers, members of the Canadian Rangers and members of the Band Council coordinated the monitoring of households to ensure they were doing well and had sufficient food supplies. If a household was in need, volunteers arranged grocery deliveries utilizing emergency COVID-19 funding. One program manager shared their experience, *“He (Chief) did a good job… he went there*,* and he gave them the food*,* even to me… Because he treated everybody like one family… no one can go to the store or anywhere*,* and they’re locked down. So*,* he gave us the food and everything”* (Participant 19). Community members expressed how they found these food deliveries essential for maintaining isolation. Additionally, these initiatives brought on a sense of unity and support, which helped the community navigate the challenges of the pandemic.

The strategy of providing food to support people staying home was not only effective in ensuring community members could obtain food, but also in controlling the spread of the virus,

*“When the pandemic hit… we ordered a bunch of food… When the numbers started rising*,* we already had [store name redacted]*,* I said I need stuff for 200 homes*,* all the little*,* just the everyday stuff… They said that’s going to take two weeks. That’s fine*,* get it… I need meat for 250 homes… Then we start getting fruit too and buying from farmers market and stuff like that so that we’re feeding people*,* so they stay home”* (Participant 7).

Although this proactive approach helped to control the spread of COVID-19 in the community, participants expressed the need for more data to support those on the front lines in future pandemics and emergencies. Having access to data from various organizations was important for creating and distributing supplies efficiently. Additionally, they highlighted the importance of leveraging existing knowledge and resources stating, *“well*,* the [store name redacted] already knows their supply and demand*,* right? So*,* you kind of leave some knowledge with certain areas*,* you just kind of give directions and ensure that adequate supplies are coming in. They know what their community demands are*” (Participant 9). Using this collaborative approach ensured that the community’s needs were met despite the challenges.

The pandemic forced many local food sources to quickly adapt their operations to continue serving the community while adhering to safety protocols. These adaptations came with their own challenges but were necessary to ensure that community members continued to have access to food supplies.

## Discussion

The COVID-19 pandemic exacerbated food insecurity globally, disproportionately affecting Indigenous communities due to their pre-existing vulnerabilities and systemic inequalities [1, 40–42]. The capacity to recover from adversity and ‘have a good life outcome despite emotional, mental, or physical distress’ [43], referred to as resilience, was demonstrated by communities like FAFN in their response to the challenges posed by the pandemic. Their ability to adapt pre-existing community-led initiatives and incorporate traditional practices played a crucial role in shaping effective strategies to address food security challenges. The emergency food initiatives that were developed and implemented during the pandemic in FAFN showcased remarkable resilience and resourcefulness, reinforcing the importance of community-driven solutions. Coordinated efforts by community members, the pandemic committee, and external support systems ensured the delivery of food supplies, while maintaining safety and limiting the spread of the virus. These initiatives underscored the importance of collaboration, data-driven decision-making, and government support in effectively managing public health crises.

An important strategy which emerged was to maintain traditional food practices as a means to improve food security during the pandemic. Several participants highlighted the importance of hunting, fishing, and gathering activities that not only provided essential nutrients, but also helped to maintain culture and mental health. These findings align with other research studies which have emphasized the importance of incorporating traditional and local food systems into programs aimed at combating food insecurity in Indigenous communities [1, 7, 17, 25, 44, 45]. Traditional food practices offer great benefits as they are important for the health and well-being of Indigenous populations [42, 46]. Maintaining and supporting these food systems ensures that communities can sustain themselves, especially during challenging times such as pandemics or other emergencies.

For communities like FAFN, which face annual environmental threats such as flooding and wildfires, emergency preparedness is critical. Community members have noted that FAFN’s experience with recurring flooding during river-ice breakups—sometimes requiring large-scale evacuations—has better equipped the community to manage issues such as food shortages during emergencies [24, 47]. Similar to the emergency funding received during the pandemic, the Emergency Management Assistance Program through ISC provides financial support for emergency planning, prevention, evacuations and responses during emergencies, such as, flooding and wildfires [48]. During the 2021 flood, ISC, in collaboration with the regional Weeneebayko Area Health Authority (WAHA), utilized this funding to support evacuations to traditional territories (i.e., bush camps) by providing transportation, supplies, and equipment that enabled community members to harvest traditional foods, such as, geese and moose [47, 48]. The strategy was used during the COVID-19 pandemic, and allowed people to travel to their camps, regardless of national travel restrictions, to ensure access to nutritious and culturally significant food. FAFN’s ability to adapt existing strategies and implement new initiatives during times of crisis highlights the importance of leveraging established capacities to enhance food security and resilience in the face of emergencies.

The pandemic committee’s emphasis on maintaining their treaty rights to hunt and fish during the pandemic illustrates the critical role of self-determination in Indigenous food security. The upholding of treaty rights reflects a broader belief among Indigenous communities in Canada and globally, where asserting sovereignty and self-determination is foundational to addressing food security and other social determinants of health [10, 49, 50]​​. However, pandemic lockdown measures and restrictions imposed during COVID-19 potentially violated these treaty rights by limiting access to traditional lands and the ability to engage in subsistence activities such as hunting and fishing, which are crucial to community food sovereignty [51]. One review of Indigenous food sovereignty initiatives across the United States of America found that the primary goals of many initiatives included “cultural preservation, health promotion, and cultural food security” [42]. These initiatives highlight the resilience of communities in asserting control over their food systems and revitalizing Indigenous knowledge and practices [42]. One review by Sampson, Cely-Santos [10] emphasized the positive impact of interventions or policies related to food sovereignty or the right to food on community-level food security and nutrition, highlighting the importance of rights-based approaches to enhance these outcomes. This aligns with the Crown-Indigenous Relations and Northern Affairs Canada’s view on food sovereignty as vital for food security, aiding Indigenous Peoples and those in the Canadian north in self-managing their food systems through existing systems, sustainable funding for community projects, innovative local food production technologies, and collaborative governance models [46]. To enhance community resilience, future studies and policies should aim to better integrate and protect treaty and other Indigenous self-governance rights during emergencies.

Promoting traditional food practices in FAFN not only enhanced food security but also increases cultural wellbeing, which is intimately tied to on-the-land activities in Canada [21]. It is essential to acknowledge that food sovereignty is deeply intertwined with cultural identity and heritage [50]. Many participants emphasized these points while discussing their engagement in traditional subsistence activities and harvesting traditional medicines. These activities are crucial not only for their continuity but also for transmitting teachings and language to the community’s youth through experiences on the land. These sentiments are echoed in the Arctic Report Card, a peer-reviewed publication by the National Oceanic and Atmospheric Administration that provides environmental insights into the Arctic’s current state compared to historical records [9, 40]. The 2021 report focused on the impact of COVID-19 on food access in Alaska, highlighting Indigenous Knowledge and recognizing its breadth, from seed sovereignty to language revitalization [9, 40]. Many participants in the study spoke of the importance of passing down this knowledge to younger generations to ensure they are prepared for future emergencies or pandemics. Prioritizing and investing in traditional and local food systems during pandemics and other emergencies allows communities to deepen their cultural connections and pass down vital knowledge to future generations.

However, despite these efforts, the pandemic also exposed and exacerbated existing vulnerabilities. The geographic isolation of Fort Albany, coupled with disrupted supply chains, highlighted the critical need for sustainable food security solutions. Existing local programs, such as, the community garden [52, 53] and Farmer’s Market became even more vital during the pandemic, but faced additional challenges like maintaining operations when workers were isolating and supply shortages. This situation mirrors findings from other remote Indigenous communities where food supply chains are fragile, and disruptions can have severe impacts [2].This points to the broader issue of labor and supply shortages and the need for robust support systems to sustain food security initiatives during the pandemic and other emergencies [42]​​. For instance, the Navajo Nation launched several initiatives to enhance food security amidst the pandemic. They revived traditional agricultural practices, such as planting heirloom seeds and cultivating ancestral lands, which not only ensured a steady food supply but also reinforced cultural heritage and resilience [54]. Similarly, in Canada, the Skidegate, an Indigenous community in British Columbia, maintained garden initiatives that included traditional medicine, fruit trees, and various vegetables [17].These efforts have not only preserved their cultural practices but also played a crucial role in mitigating the impact of job losses during the pandemic. By leveraging these garden projects, the community was able to provide 90 families with weekly food security boxes, which included a combination of traditional foods and other nutritious items [1, 17]. This initiative has been instrumental in ensuring food security and promoting health and well-being among community members during challenging times [1, 17].

FAFN’s response to food insecurity during the COVID-19 pandemic underscores the importance of integrating community-based knowledge into modern response strategies for pandemics and other emergencies. This study provides valuable lessons for rural-and-remote communities, particularly Indigenous communities facing similar challenges. These findings emphasize the need for culturally relevant and community-driven approaches in crisis management. The pandemic committee, composed of diverse members including individuals from various sectors of the community—such as those with expertise in health, business, and community initiatives—played a pivotal role in coordinating and implementing responsive strategies. Their collective knowledge and collaboration ensured that interventions were not only effective but also culturally appropriate and sustainable. These insights contribute to the growing body of literature on community-driven responses to pandemics and emergencies. Recognizing and supporting these elements during emergencies and beyond will contribute to the health, well-being, and resilience of Indigenous populations, fostering stronger, more resilient communities equipped to face future challenges.

This study, while providing valuable insights into the food security strategies of FAFN during the COVID-19 pandemic, has some limitations. Firstly, the data were gathered from a limited number of participants who were either on the pandemic committee or managed programs. This selective approach ensured that the information came from key participants directly involved in decision-making and program delivery, adding depth and relevance to the findings. Moreover, the unique experiences and perspectives of these individuals offer a nuanced understanding of the community’s strategies. Furthermore, the study’s focus on FAFN provides locally significant insights. While the geographic, cultural, and social characteristics of FAFN are unique, the lessons learned through this research can inform approaches in other Indigenous communities, highlighting how tailored strategies can effectively address local needs.

## Conclusion

The present article showcases several examples of FAFN’s adaptability and resourcefulness in addressing food security challenges during the COVID-19 pandemic, highlighting the successes of community-led responses. By utilizing traditional practices and other approaches, while leveraging local data, the community addressed some of the pandemic’s impacts on food security. These insights contribute to a broader understanding of food security in Indigenous contexts, emphasizing the need for holistic and inclusive approaches that respect Indigenous knowledge and practices. Emphasizing traditional and local food systems enhances emergency preparedness, allowing communities to be better equipped to handle disruptions caused by pandemics and other crises. This underscores the importance of culturally relevant, community-driven strategies in addressing food security, particularly during pandemics and emergencies, and emphasizes the benefits of incorporating Indigenous knowledge to tackle contemporary challenges.

The experiences of FAFN community members offer valuable lessons for policymakers and provides insights for other communities. Policies and programs aimed at supporting Indigenous food sovereignty should be developed in collaboration with Indigenous leaders and communities. This collaborative approach ensures that the needs and perspectives of Indigenous peoples are respected and addressed. Governments and organizations must also allocate adequate resources and support to facilitate the implementation and sustainability of these initiatives. By examining these innovative strategies and programs, researchers and policymakers can better support and enhance the resilience of communities in the face of ongoing and future challenges.

## Data Availability

In accordance with the principles of Ownership, Control, Access, and Possession (OCAP^®^), all datasets generated and/or analyzed during this study are the intellectual property of Fort Albany First Nation. As such, these datasets are not publicly available. Any inquiries regarding the study or requests for further information should be directed to the corresponding author.
